# Enhancing Laboratory Response Network Capacity in South Korea

**DOI:** 10.3201/eid2313.170348

**Published:** 2017-12

**Authors:** J. Todd Parker, Ann-Christian Juren, Luis Lowe, Scott Santibañez, Gi-eun Rhie, Toby L. Merlin

**Affiliations:** Centers for Disease Control and Prevention, Atlanta, Georgia, US A (J.T. Parker, A.-C. Juren, L. Lowe, S. Santibañez, T.L. Merlin);; Korea Centers for Disease Control and Prevention, Osong, South Korea (G.-e. Rhie)

**Keywords:** Laboratory Response Network, South Korea, bioterrorism and preparedness, preparedness, global health security

## Abstract

Laboratory Response Network (LRN) laboratories help protect populations from biological and chemical public health threats. We examined the role of LRN biological laboratories in enhancing capacity to detect and respond to public health infectious disease emergencies in South Korea. The model for responding to infectious disease emergencies leverages standardized laboratory testing procedures, a repository of standardized testing reagents, laboratory testing cooperation among hospital sentinel laboratories and reference laboratories, and maintenance of a trained workforce through traditional and on-demand training. Cooperation among all network stakeholders helps ensure that laboratory response is an integrated part of the national response. The added laboratory testing capacity provided by the US Centers for Disease Control and Prevention LRN assets helps protect persons who reside in South Korea, US military personnel and civilians in South Korea, and those who reside in the continental United States.

The US Centers for Disease Control and Prevention (CDC), in cooperation with the Federal Bureau of Investigation (FBI) and the Association of Public Health Laboratories, developed the Laboratory Response Network (LRN) as part of the strategic infrastructure that keeps the United States safe from intentional and naturally occurring public health threats (*1*). The LRN has a broad capacity to detect biological and chemical public health threats. LRN laboratories that detect and identify biological threat agents, such as *Bacillus anthracis*, ricin toxin, or variola virus, are referred to as LRN-B laboratories; those that detect chemical agents are called LRN-C laboratories. The LRN-B comprises clinical, food, veterinary, environmental, and agricultural laboratories that work together to detect and identify agents that have historically been considered potential weapons of mass destruction (*2*). The LRN-B currently has 139 reference microbiology laboratories; ≈100 laboratories are in the United States, and member laboratories (which have access to LRN-B assets) are in Canada, Australia, and South Korea (Figure 1). Increasing the number of LRN-B laboratories worldwide can help countries more rapidly detect, respond to, and contain public health emergencies at their source and thereby enhance global health security.

**Figure 1:**
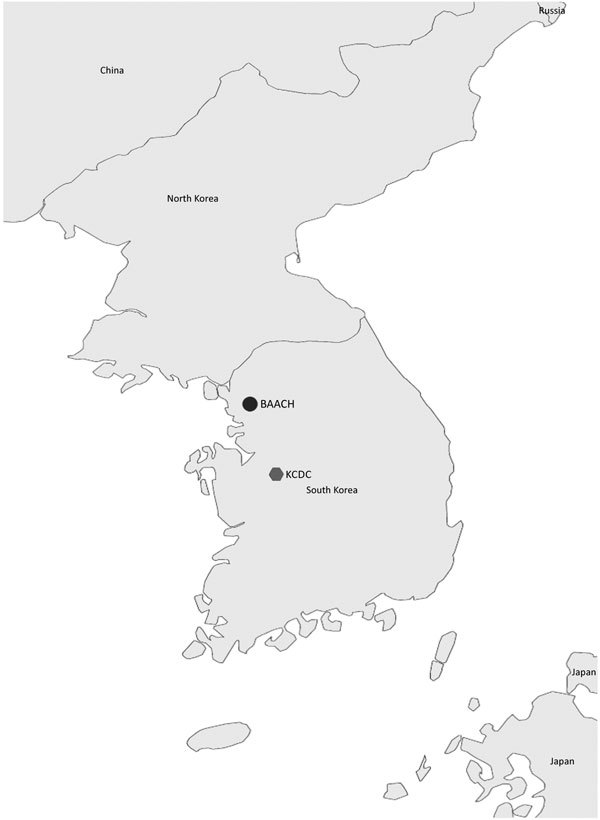
Locations of Laboratory Response Networks in South Korea. BAACH, Brian Allgood Army Community Hospital, US Army Yongsan Garrison, Seoul; KCDC, Korea Center for Disease Control, Osong.

South Korea (also called the Republic of Korea) is geographically situated in a region at high risk for a state-sponsored release of biological or chemical agents (*3*,*4*). In addition to the estimated 50 million South Korea residents, >28,500 US military personnel and 136,600 US civilians live and work there (*5*). Risks for a deliberate biological agent release in South Korea affect the local population (*1*), US military personnel and civilians who live and work on the Korean peninsula (*2*), and the population of the continental United States through imported cases and secondary transmission (*3*).

The establishment of LRN-B laboratories in South Korea enables these laboratories to access the standardized LRN testing procedures and reagents. This access helps leverage US response assets in the event of a biological agent release, thereby assisting in the protection of all 3 populations described above. We describe the development of the LRN model in the United States (*1*), how the US LRN model works by using a 3-tiered system (*2*), and collaborative efforts to enhance international–US CDC LRN capacity in South Korea (*3*). 

## Development of the LRN Model

In 1999, the US LRN was founded as a collaboration among CDC, the Association for Public Health Laboratories, and the FBI. The initial focus of the LRN-B centered on identification of potential bioterrorism pathogens (*6*). The LRN subsequently developed into an integral component of detection and response to outbreaks of severe acute respiratory syndrome (2003), monkeypox (2003), Middle East respiratory syndrome (MERS; 2013), Ebola (2014–2015), and Zika virus infection (2016).

LRN-B laboratories use a 3-tiered system. The first tier comprises ≈5,000 sentinel microbiology laboratories, located mainly in hospitals and clinics. The role of a sentinel laboratory is not to confirm the identity of a particular suspected bioterrorism pathogen but rather to identify frequently encountered bacteria with similar culture characteristics or to refer the specimen to an LRN reference-level microbiology laboratory (*7*,*8*).

The second tier of the LRN-B comprises reference-level microbiology laboratories, typically state, city, or local public health laboratories, or military, veterinary, and agriculture laboratories (*9*). LRN-B reference laboratories follow testing algorithms to rapidly identify specific presumed and confirmed bioterrorism pathogens.

The third tier of the LRN-B comprises agencies such as CDC and the US Army Medical Research Institute of Infectious Diseases. The LRN reference-level laboratories can refer isolates that require further characterization to these laboratories.

In addition to the 3-tiered system, the LRN program office at CDC manages several network assets that facilitate national preparedness. These assets include secure access to standardized pathogen-detection procedures, a repository of quality pathogen-detection reagents, a robust proficiency-testing program, secure laboratory communication and reporting processes, and expertise in Emergency Use Authorizations for emergency response (*10*). The Food and Drug Administration (FDA) can authorize (FDA approval) pathogen-detection testing of human clinical specimens for the duration of a declared emergency. The LRN program office has worked with FDA for Emergency Use Authorizations deployment and to predeploy assays for severe acute respiratory syndrome, MERS, Ebola, and Zika virus infection.

During the US LRN membership enrollment process, foreign and domestic laboratories self-determine the extent of the LRN-B testing portfolio that their laboratory will implement, based on their resources and the threats that they are most likely to encounter. LRN member laboratories are tested on their proficiency to respond to test challenges, based on self-reported biological agent–specific testing capability. In addition, training in regulatory compliance and documentation is essential for those laboratories that ship pathogens and pathogen-derived material used for proficiency testing and specimen/sample referral.

## Building International–US CDC LRN Capacity in South Korea

In 2011, the CDC LRN program office and the US Department of Defense began establishing an international–US CDC LRN member laboratory in South Korea. Since 2002, the Korea Centers for Disease Control and Prevention (KCDC) had already been operating a laboratory response network similar in structure to that of the US LRN, using its own procedures and reagents. However, establishing a US LRN presence in South Korea enabled its use of US LRN-B procedures and reagents, in addition to other US LRN assets, which are accessible only to US LRN member laboratories.

To establish an international–US LRN-B presence, KCDC and the US Department of Defense identified 2 locations: 1 on a joint US/South Korea military facility (the Brian Allgood Army Community Hospital [BAACH] at Yongsan US Army Garrison in Seoul) and 1 at a South Korea public health facility (KCDC Division of High-Risk Pathogen Research in Osong). The BAACH functions as a sentinel and reference-level laboratory for the base personnel and their families; the Division of High-Risk Pathogen Research is a public health laboratory within the KCDC.

**Figure 2:**
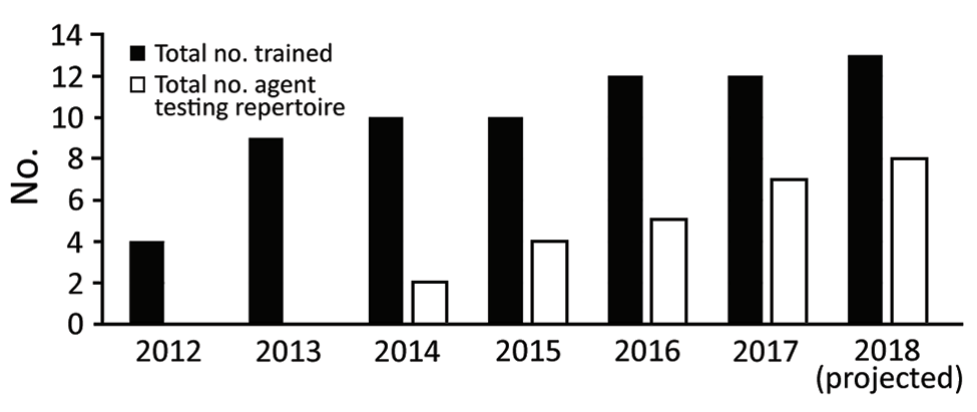
Training and testing capacity building for Laboratory Response Networks in South Korea. Training and expanded testing capability are synergistic. Total number trained indicates the number of laboratory personnel from the Brian Allgood Army Community Hospital, US Army Yongsan Garrison, and from the Korea Centers for Disease Control and Prevention facility trained on either rapid diagnostics or confirmatory conventional microbiology. Total number of agents in testing repertoire indicates the biological threat agent testing capability when Laboratory Research Network procedures, as determined by proficiency testing, are used.

The initial steps for adding the US LRN capability were provision of training for confirmatory procedures that use standard culture and biochemical techniques and rapid procedures for presumptive identification that use molecular and antigen-detection technologies. Critical portions of training documents were translated into Korean. These portions included laboratory job aids and specific laboratory procedures such as data interpretation and assay limitations. Biological select agents and toxins have the potential to pose a severe threat to public, animal, or plant health or to animal or plant products. The LRN program office worked with the US LRN Army Medical Command partners to select laboratory personnel who were fluent in the Korean language, security-risk assessment (SRA) approved (i.e., authorized to directly handle cultures of select agent organisms), and able to take the course themselves and partner with KCDC laboratory course students at the LRN confirmatory microbiology course to assist with language barriers. The logistics for an LRN Conventional Methods course are complicated. Only students with prior SRA approval from the FBI may directly handle cultures or material considered a select agent, and the class may be held only in a select agent registered laboratory (*11*). Using SRA-approved, Korean-fluent US Army laboratory course students enabled the LRN course trainers to provide one-on-one translation and direct laboratory observance of bacterial select agent culture characteristics by the KCDC course students, without compromising compliance with US select agent regulations. The LRN program office selected the US Hawaii public health laboratory to host the LRN Conventional Methods training course. This laboratory was chosen because it is an LRN-member laboratory, uses cultures of select agent bacteria, is registered to handle select agent pathogens, and is relatively near South Korea and the US mainland. During 2012–2013, two training courses were completed by 9 persons from the Korea-based International–US CDC LRN laboratories (Figure 2). In 2013, the Yongsan Garrison facility hosted an LRN Rapid Methods course, which focuses on sample processing, and a Rapid Molecular/Antigen Detection course for co-participating US Army and KCDC students. The Rapid Methods course did not use select agents during the training, which simplified the importation of materials and course implementation.

In 2016, a CDC team including members of the LRN program office, an infection prevention practitioner, a CDC poxvirus subject matter expert, and a high containment laboratory (HCL) manager traveled to South Korea to help develop training similar to that used at other LRN-B facilities to further enhance emerging infectious diseases (EID) response capability. At the KCDC HCL, the CDC poxvirus subject matter expert and CDC HCL manager helped develop training for safe HCL entry and exit and man-down emergency HCL exit training. Emergency man-down training involves a simulated emergency involving a person who needs immediate medical intervention because of a life-threatening incident (e.g., collapsing while at work). In such a situation, a person might need to be extracted from an HCL facility as rapidly as possible without compromising overall safety.

The BAACH hospital and laboratory are unique to the LRN because they function as a primary care hospital and as both sentinel-level and reference-level LRN laboratories. To assist BAACH in preparedness for an EID outbreak, members of the LRN program office and a CDC infection prevention practitioner held discussion-based EID training and assessment of best practices and information sharing for the medical and laboratory staff. The EID training for BAACH followed the same model as the US Ebola Risk Assessment training. This format, which is the usual type of training used in other LRN-B laboratories, integrates hospital, LRN sentinel-level laboratory, and LRN reference-level laboratory personnel to understand roles, responsibilities, and communication among stakeholders. The discussion was based on review of the laboratory component of a 2015 MERS outbreak in South Korea and hospital laboratory preparedness for an EID event.

## Leveraging Technology to Enhance Laboratory Capacity

**Figure 3:**
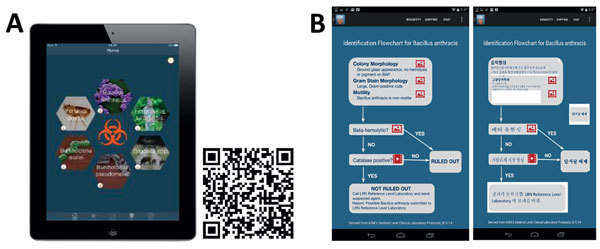
On-demand training tools sustain and enhance laboratory pathogen identification as part of the Laboratory Research Network. A) The Laboratory Research Network Rule-Out and Refer mobile application, available for download on Apple tablets via QR code or the Apple App store. B) Flowcharts provide easy agent-specific rule-out and refer information, including images and videos in English and Korean.

A valuable component of this collaborative effort is the leveraging of technology for continuing education. On-demand technology resources include offering additional training resources to LRN-B domestic and international partners in the form of a mobile smartphone or tablet application (app) and online proficiency assessments. Our standard for on-demand training is the LRN Rule-Out and Refer mobile app (Figure 3). This app is a support tool for sentinel laboratories, providing integrated agent-specific, bacterial biological threat rule-out and refer testing flowcharts and additional information to assist the laboratorians (*11*). The testing algorithms derive from American Society for Microbiology guidelines and are available for tablet devices, which can be sequestered for in-laboratory use only and can access updates by wireless connection (*2*). This mobile app can work as a quick reference and as a formative training tool. The bacterial agent–specific rule-out and refer flowcharts in the app have been translated into Korean.

Recently, the LRN-B program office added virtual online proficiency assessments as a complement to existing training resources. The proficiency assessments are used during hands-on laboratory courses, to reinforce learning, and as an on-demand informative training tool. The proficiency assessments are accessed through a secure online link that provides instantaneous feedback to participants and allows for tailored knowledge remediation from the training providers. These tools were first used in the 2016 Conventional Methods training classes and are currently being translated into Korean. The content of the proficiency assessments are expanding to include content for the rapid presumptive procedures and other agent-specific EID training for participating LRN laboratories. The intrinsic value of these technologies as training tools is increased by their accessibility and versatility, providing optimum functionality in the global context of the LRN-B and the goal of maintaining a trained workforce.

## Future Steps for Increasing Capacity

Enhancing LRN capacity in South Korea helps protect the population of South Korea, US military personnel and civilians in South Korea, and the population of the continental United States, and thereby enhances global health security. In addition, South Korea is geographically and technologically poised to serve as a hub for public health functions in Southeast Asia. These functions could include enhancing infectious disease detection capability and providing leadership on global health security initiatives. Continued collaboration with partners in South Korea provides a mechanism for rapidly disseminating processes, procedures, and reagents before and during a public health crisis. The LRN-B collaboration in South Korea and the use of standardized procedures, which lead to an added assurance of laboratory results, could provide a catalyst for engaging partners in other Southeast Asia countries. Combined, these partnerships and sharing of information benefit the public health for residents of South Korea and for US personnel serving in Southeast Asia.
